# Age-Related Changes in Item Responses to the Patient Health Questionnaire-9: Evidence From the National Health and Nutrition Examination Survey

**DOI:** 10.3389/fpsyt.2020.00723

**Published:** 2020-07-22

**Authors:** Shinichiro Tomitaka, Yohei Kawasaki, Kazuki Ide, Maiko Akutagawa, Yutaka Ono, Toshiaki A. Furukawa

**Affiliations:** ^1^Department of Mental Health, Panasonic Health Center, Tokyo, Japan; ^2^Department of Health Promotion and Human Behavior, Kyoto University Graduate School of Medicine/School of Public Health, Kyoto, Japan; ^3^Clinical Research Center, Chiba University Hospital, Chiba, Japan; ^4^Uehiro Research Division for IPS Cell Ethics, Center for iPS Cell Research and Application, Kyoto University, Kyoto, Japan; ^5^Department of Drug Evaluation and Informatics, School of Pharmaceutical Sciences, University of Shizuoka, Shizuoka, Japan; ^6^Center for the Development of Cognitive Behavior Therapy Training, Tokyo, Japan

**Keywords:** age, depression, depressive symptom severity, Patient Health Questionnaire-9, depression screening tools, item response, ordinal scale

## Abstract

**Background:**

Epidemiological studies have shown that total scores in depression screening scales change with age, but the mechanism underlying these age-related changes remains unclear. Previous research has indicated that item responses in depression screening scales exhibit characteristic distributions in the general population. We analyzed Patient Health Questionnaire-9 (PHQ-9) data from a representative survey conducted in the USA, to determine how the response pattern for each item changed with age and whether the pattern of responses contributed to age-related changes in total scores.

**Methods:**

We analyzed PHQ-9 data for 17,274 participants in the 2011–2016 National Health and Nutrition Examination Survey. The PHQ-9 allows respondents to self-rate the frequency of depressive symptoms using a four-point scale ranging from “not at all” to “nearly every day”.

**Results:**

The lines for all nine item responses followed the same characteristic pattern across all age groups, which was marked by intersection at a single point between “not at all” and “several days” and parallel patterns between “several days” and “nearly every day” on a logarithmic scale. The probability of “nearly every day” showed a reverse U-shaped pattern, in that it was low from 12–29 years, increased during 30–50 years, and then decreased at ≥60 years. The age-related change in the probability of a response of “nearly every day” coincided with the trajectory of the PHQ-9 total scores.

**Conclusions:**

This study demonstrated that item responses for the PHQ-9 followed a similar mathematical pattern across the adult lifespan. Moreover, our findings suggested that the probability of a response of “nearly every day” played an important role in age-related changes in PHQ-9 total scores across adulthood.

## Introduction

Depression is a common mental disorder and a leading cause of disability worldwide (1). It is clinically important to identify the age at which people are susceptible to becoming prone to clinical depression (2). Because the diagnosis of depression is based on the severity of depressive symptoms, numerous studies have attempted to elucidate age-related changes in such symptoms using a variety of screening tools for depression (2–6). However, findings regarding age-related changes in depressive symptoms have been inconsistent (2–7).

The nine-item Patient Health Questionnaire (PHQ-9) is based on the criteria for major depression stated in the Diagnostic and Statistical Manual of Mental Disorders (8) and widely used as a screening tool for depression worldwide (9, 10). It is noteworthy that population-based studies using the PHQ-9 and its related versions have shown inconsistent evidence in age-related changes in total scores. The National Health and Nutrition Examination Survey (NHANES) and Behavioral Risk Factor Surveillance Survey, conducted in the USA, showed that the trajectory of total scores followed a reverse U-shaped pattern, increasing from young to middle adulthood and decreasing during older age (11, 12). In contrast, population-based studies conducted in Germany and India showed that total PHQ-9 scores increased with age (13–16), while Chinese and Canadian studies demonstrated that total scores decreased with age (17, 18). The reason for the inconsistent evidence regarding age-related changes in total scores on the PHQ-9 remains unclear. To understand this inconsistency, it is necessary to clarify the basis of age-related changes in PHQ-9 total scores.

In the general population, item responses in self-rated depression scales have recently been reported to exhibit a characteristic distribution (19–21). In an analysis of PHQ-9 data from the NHANES in the USA, we found that item responses for the PHQ-9 exhibited a common pattern across all nine items (20). Specifically, lines of item responses crossed at a single point between “not at all” and “several days” and showed a parallel pattern for the remaining response options on a logarithmic scale (20). This common characteristic pattern has been replicated for PHQ-8 data from the Behavioral Risk Factor Surveillance Survey in the USA (21), data for the Center for Epidemiologic Studies Depression Scale (CES-D) from the Irish Longitudinal Study on Ageing (22) and the Japanese Active Survey of Health and Welfare (19), and data for the six-item Kessler Psychological Distress Scale (K6) from the National Survey of Midlife Development* *in the USA (23).

The mathematical pattern of item responses to depressive symptom items in a general population is important for some reasons. Currently, statistical models that assume normal distributions are often used to analyze data regarding the PHQ-9 (24). However, if the observed item responses on the PHQ-9 considerably differ from normal distributions, such statistical models would require consideration. Furthermore, the mathematical pattern of responses to depressive symptom items will help understand how the degree of depressive symptoms is distributed in a general population.

**Figure 1:**
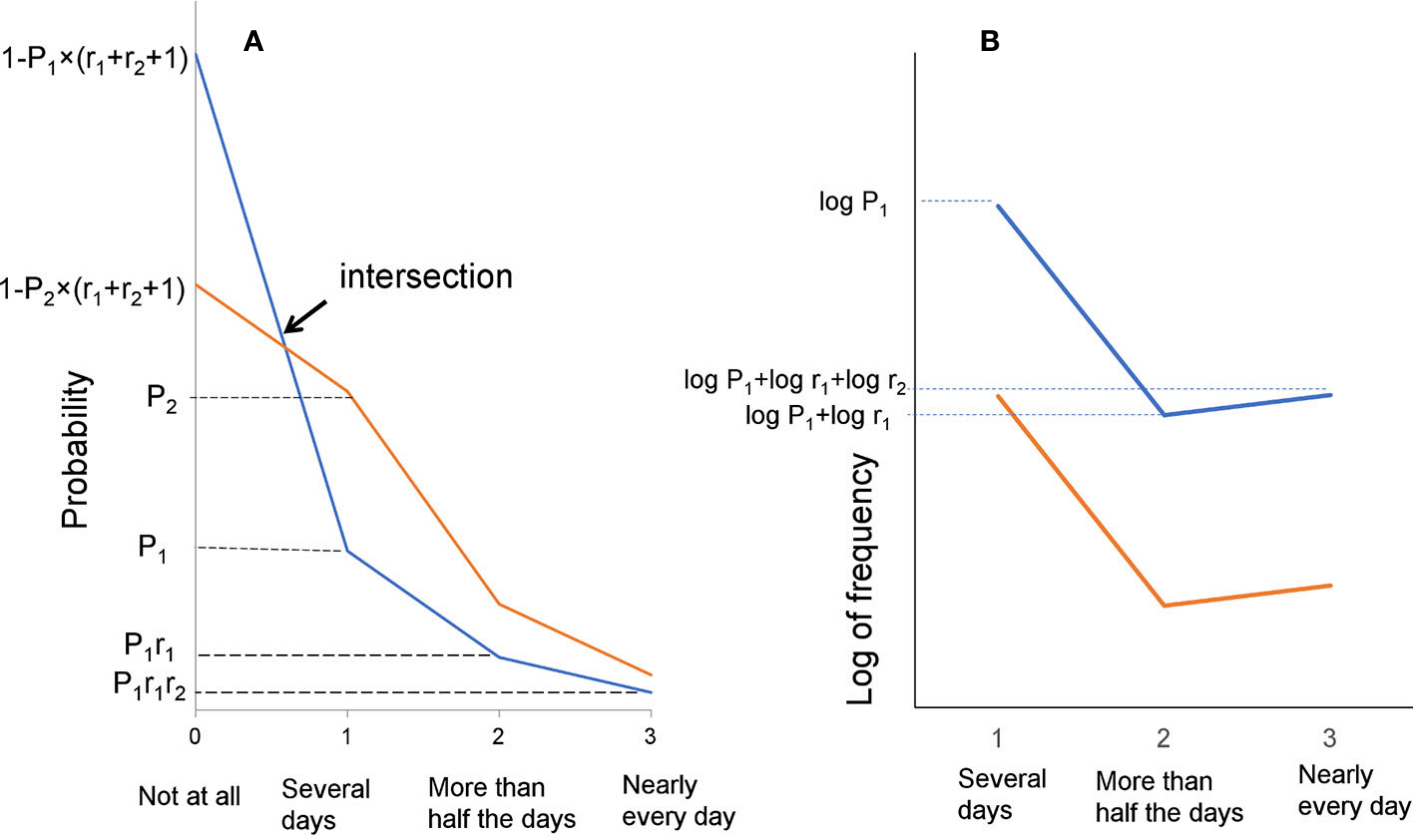
The model for responses to depressive symptom items on the PHQ-9. **(A)** This mathematical model is based on the results that the ratios of “several days” to “more than half the days,” and “more than half the days” to “nearly every day,” are similar among all items. **(B)** A model of the parallel pattern between “several days” and “nearly every day” on a logarithmic scale.

Previous studies have shown that the ratios of “several days” to “more than half the days,” and “more than half the days” to “nearly every day,” were similar among all items (20). Based on these findings, we proposed a model for responses to depressive symptom items on the PHQ-9 (25). As shown in **Figure 1A**, when the probability of “several days” is presented as Pi (i = item number) and the ratio of “more than half the days” to “several days” and “nearly every day” to “more than half the days” are presented as two constants, r_1_ and r_2_, the probabilities of “several days,” “more than half the days,” “nearly every day,” and “not at all” are expressed as Pi, Pir_1_, Pir_1_r_2_, and 1 – Pi × (1 + r_1_ + r_1_r_2_), respectively. Mathematically, if the two constants r_1_ and r_2_ are the same for all nine items, all lines for the items cross at a single point between “rarely” and “several days” (**Figure 1A**), and exhibit a parallel pattern from “several days” to “nearly every day” on a logarithmic scale (**Figure 1B**) (25).

As total scores are calculated as the sum of all item scores, patterns in the responses for each item have been suggested to contribute to age-related changes in total scores. In fact, in our previous study, age-related changes in total CES-D scores were attributed to age-related changes in common response patterns for all items (26). However, to our knowledge, little is known about how item response patterns for the PHQ-9 contribute to age-related changes in total scores.

In the current study, we examined how item response patterns for the PHQ-9 changed with age. We sought to confirm whether item responses for the nine items followed a characteristic pattern across all age groups and item scores were responsible for the reverse U-shaped pattern of total PHQ-9 scores; thereafter, we aimed to determine whether age-related changes in total PHQ-9 scores occurred because of age-related changes in specific response options for items.

This study analyzed PHQ-9 data from the NHANES, which is a national survey designed to assess health and nutritional status in the USA (27). PHQ-9 data from the NHANES are suitable for use in the identification of the aforementioned pattern, because of the large sample size.

## Material and Methods

### Dataset

The study used NHANES 2011–2016 data (28). The NHANES is a continuous cross-sectional survey of a nationally representative sample of noninstitutionalized civilian US citizens. The NHANES uses a multistage design to select participants, and the survey includes a household interview and an examination carried out in a mobile examination center. In total, 43,090 participants were selected for the 2011–2016 NHANES. Of these participants, approximately half were aged 18 years or older and completed the PHQ-9 questionnaire. The NHANES sample included in the current study consisted of 17,274 respondents [aged 19–29 years, *N* = 3,351 (men: *n* = 1,683); aged 30–39 years, *N* = 2,434 (men: *n* = 1,244); aged 40–49 years, *n* = 2,411 (men: *n* = 1,130); aged 50–59 years, *n* = 2,383 (men: *n* = 1,147); aged 60–69 years, *n* = 2,542 (men: *n* = 1,246); and aged ≥70 years, *n* = 2,310 (men: *n* = 1,126)]. The sociodemographic characteristics of the 2011–2016 NHANES sample are reported in detail elsewhere (29).

As the institutional review board at the Panasonic Health Center does not regard secondary analysis of deidentified public data as human subject research, this study was exempt from the need for ethical approval. All NHANES participants provided written informed consent at the time of data collection.

### Measures

The 2011–2016 NHANES used the PHQ-9 to evaluate the severity of depressive symptoms. The PHQ asks respondents to report the frequency of depressive symptoms over the preceding 2 weeks using the following scale: 0 = not at all, 1 = several days, 2 = more than half the days, and 3 = nearly every day (**Supplementary Table 1**). Total PHQ-9 scores range from 0 to 27.

### Analysis

Participants were classified into the following age groups: 18–29 years, 30–39 years, 40–49 years, 50–59 years, 60–69 years, and ≥70 years. We compared total PHQ-9 scores with each item score according to age group and determined whether the nine items of the PHQ-9 followed the same reverse U-shaped pattern as that of the total PHQ-9 score across adulthood. Further, to confirm that the item responses followed the same characteristic pattern for the nine items across all age groups, the respective line graphs were evaluated, with line graphs created for each age group. In the previous study, the lines for all nine item responses in the PHQ-9 exhibited a parallel pattern from “several days” to “all of the time” on a logarithmic scale (20). Therefore, responses between “several days” and “nearly every day” were analyzed using logarithmic scales.

In addition, to elucidate age-related changes in item responses for the nine items, we calculated the averaged probability of “not at all,” “several days,” “more than half the days,” and “nearly every day” across the nine items and examined the trajectory of the averaged probability of each answer option across age groups. Theoretically, the mean item scores are calculated from the probability of each item response option. Let the probability values for responses of “several days,” “more than half the days,” and “nearly every day” for the nine items be P_1_, P_2_, and P_3_, respectively. On an ordinal scale (i.e., 0-1-2-3), the mean item scores for the nine items were expressed as P_1_ + 2P_2_ + 3P_3_. Analyses were performed using JMP version 11.2.1 software (SAS Institute, Cary, NC).

## Results

### Age-Related Changes for Each Item Score and Total Scores Across Adulthood

Participants who did not choose any of the four options for all nine items were excluded from the analysis. Therefore, the final sample in the analysis included data for 15,431 participants in the 2011–2016 NHANES. The excluded sample consisted of 1,843 (10.7%) individuals.

**Figure 2:**
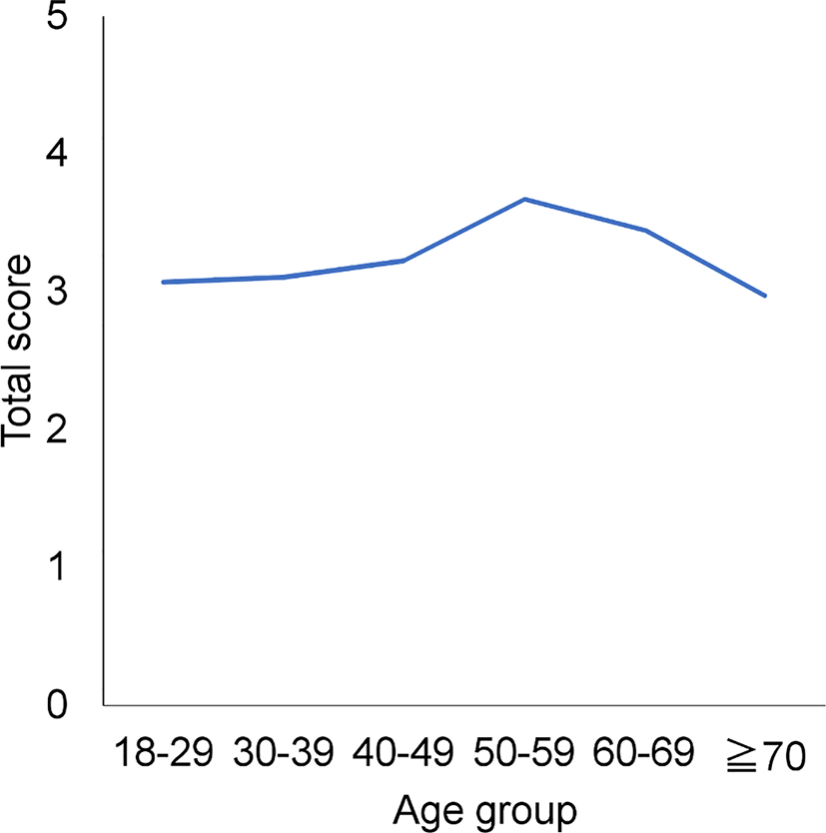
Age-related changes in each item score and total scores across adulthood. Total PHQ-9 scores exhibited a reverse U-shaped pattern, increasing from 18–29 to 50–59 years and decreasing at ≥60 years.

The total PHQ-9 scores exhibited a reverse U-shaped pattern, increasing from 18–29 years to 50–59 years and decreasing at ≥60 years (**Figure 2**), consistent with the previous report (12). Analysis of each PHQ-9 item score showed that those for seven of the nine items were highest at 50–59 years, indicating that the reverse U-shaped pattern applied to almost all item scores (**Table 1**). Scores for two items (i.e., “low energy” and “psychomotor”) were not highest at 50–59 years; however, they were second highest at this age, exhibiting a similar tendency toward a reverse U-shaped pattern.

**Table 1:** Mean item scores for the nine items according to age group.

Item	Age group					
	18–29	30–39	40–49	50–59	60–69	≥70
Anhedonia	0.354	0.348	0.377	**0.462**	0.445	0.375
Depressed	0.304	0.320	0.356	**0.409**	0.402	0.307
Sleep problems	0.597	0.569	0.581	**0.691**	0.672	0.555
Low energy	0.726	0.753	0.760	0.777	0.721	**0.785**
Appetite loss	0.399	0.415	0.391	**0.454**	0.385	0.297
Self-esteem	0.244	0.264	0.269	**0.311**	0.264	0.192
Concentration	0.249	0.249	0.279	**0.299**	0.283	0.236
Psychomotor	0.139	0.143	0.166	0.196	**0.213**	0.168
Suicidal ideation	0.049	0.046	0.047	**0.073**	0.065	0.058
Mean	0.340	0.345	0.358	**0.408**	0.383	0.330

The highest mean scores among all age groups are in bold.

### Pattern of Item Responses for the Nine Items

**Figure 3:**
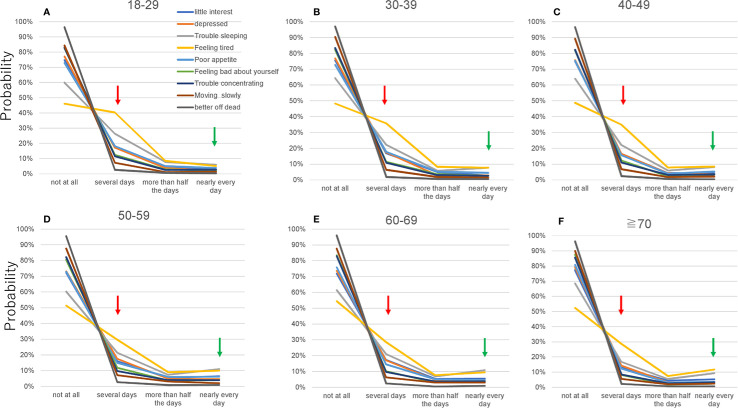
Item responses for all nine items by age group. **(A)** 18–29, **(B)** 30–39, **(C)** 40–49, **(D)** 50–59, **(E)** 60–69, and **(F)** ≥70 years. Red arrows point to the probability of a response of “several days,” and green arrows point to the probability of a response of “nearly every day.” Although item responses for the nine items demonstrated a common pattern across all age groups, the graphs of the item responses changed slightly with age. As the red arrows indicate, the probability of a response of “several days” for the nine items decreased with age. Conversely, as the green arrows indicate, the probability of a response of “nearly every day” increased slightly from 18–29 to 50–59 years **(A–D)** and was unclear at ≥60 years **(D–F)**.

To confirm that item responses for the nine items followed the same characteristic pattern, the patterns of responses were evaluated graphically for each age group (**Figure 3**). The item responses for all items demonstrated a common characteristic pattern across all age groups (**Figure 3**). The lines for the nine items intersected at a single point between “not at all” and “several days.” However, the line for “low energy” from 18–29 to 40–49 years appeared to intersect distal to the point of convergence. Conversely, the lines from “several days” to “nearly every day” were regularly skewed toward “several days”.

**Figure 4:**
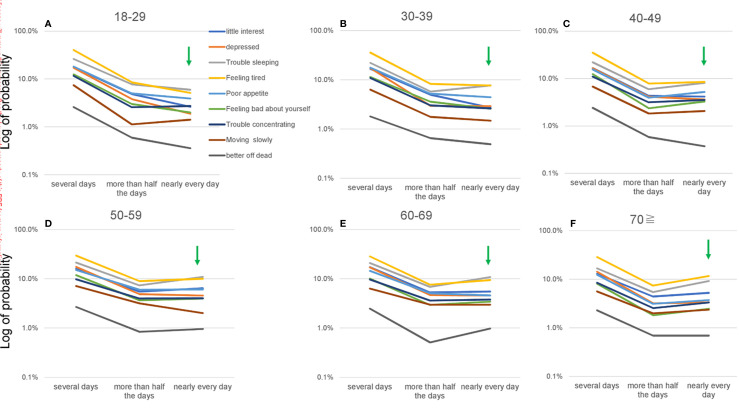
Item responses for “several days” to “nearly every day” for all nine items according to age group, using a logarithmic scale. **(A)** 18–29, **(B)** 30–39, **(C)** 40–49, **(D)** 50–59, **(E)** 60–69, and **(F)** ≥70 years. Green arrows show the probability of a response of “nearly every day.” While item responses from “several days” to “nearly every day” appeared to follow a parallel pattern for all nine items across all age groups. The log of the probability of a response of “nearly every day” increased slightly from 18–29 to 50–59 years **(A–D)** and was unclear at ≥60 years **(D–F)**.

On a logarithmic scale, item responses for the nine items appeared to follow a parallel pattern between “several days” and “nearly every day” across all age groups (**Figure 4**). Some of the lines (i.e., “better off dead” between “more than half the days” and “nearly every day” at 40–49 years) were further from parallel.

Although the item responses followed a common characteristic pattern across all age groups, the graphs of the responses appeared to change slightly with age (**Figure 3**). Focusing on the probability of responses of “several days” and “nearly every day,” we evaluated age-related changes for the items. The probability of a response of “several days” decreased with age for the nine items. Conversely, the probability of a response of “nearly every day” increased considerably from 18–29 to 50–59 years (**Figures 3A–D**) and was unclear at ≥60 years (**Figures 3D–F**).

On a logarithmic scale, the slopes of the lines for the nine items appeared to change slightly according to age (**Figure 4**). The log of probability for a response of “nearly every day” increased slightly from 18–29 to 50–59 years (**Figures 4A–D**) and was unclear at ≥60 years (**Figures 4D–F**), which was consistent with the results of standard scales (**Figure 3**).

To elucidate age-related changes in item responses precisely, we calculated the probability of each response option for the nine items for each age group (**Table 2**). Consistent with the results of graphical analysis, the probability of a response of “several days” decreased with age for seven of the nine items. Conversely, the probability of a response of “nearly every day” and “nearly every day” increased considerably from 18–29 to 50–59 years and decreased between 50–59 years and ≥70 years for six of the nine items.

**Table 2:** Probability of each response option for the nine items according to age group.

Item	Response option	Age Groups					
		18–29	30–39	40–49	50–59	60–69	≧70
Anhedonia	Not at all	**74.8%**	75.6%	74.9%	72.1%	71.8%	77.4%
	Several days	**17.8%**	16.8%	16.6%	15.9%	17.3%	13.0%
	More than half the days	4.9%	5.0%	4.3%	**5.5%**	5.3%	4.4%
	Nearly every day	2.6%	2.7%	4.1%	**6.4%**	5.5%	5.2%
Depressed	Not at all	**77.1%**	76.7%	75.7%	73.1%	73.6%	79.2%
	Several days	17.2%	17.4%	16.5%	**17.5%**	17.1%	14.3%
	More than half the days	3.8%	3.0%	4.1%	**4.9%**	4.7%	3.2%
	Nearly every day	1.9%	2.9%	3.6%	**4.6%**	4.6%	3.3%
Sleep problems	Not at all	60.0%	64.4%	64.0%	60.3%	61.4%	**68.5%**
	Several days	**26.3%**	22.1%	22.0%	21.4%	20.9%	16.8%
	More than half the days	**7.7%**	5.8%	5.9%	7.3%	6.8%	5.5%
	Nearly every day	6.0%	7.7%	8.1%	**11.0%**	10.9%	9.3%
Low energy	Not at all	46.1%	48.3%	48.7%	51.3%	**54.4%**	52.3%
	Several days	**40.3%**	35.8%	35.0%	29.7%	28.5%	28.6%
	More than half the days	8.5%	8.3%	7.8%	**9.0%**	7.6%	7.4%
	Nearly every day	5.1%	7.7%	8.5%	10.0%	9.5%	**11.7%**
Appetite loss	Not at all	72.9%	72.6%	75.3%	72.8%	75.8%	**80.8%**
	Several days	**18.2%**	17.7%	15.5%	15.1%	14.5%	12.3%
	More than half the days	5.0%	5.2%	3.9%	**6.0%**	5.1%	3.1%
	Nearly every day	3.9%	4.4%	5.2%	**6.1%**	4.6%	3.7%
Self-esteem	Not at all	82.5%	82.4%	82.0%	80.5%	83.5%	**87.6%**
	Several days	**12.5%**	11.4%	12.3%	11.9%	10.1%	8.1%
	More than half the days	3.0%	3.6%	2.4%	**3.7%**	3.0%	1.8%
	Nearly every day	2.0%	2.6%	3.3%	**4.0%**	3.5%	2.5%
Concentration	Not at all	83.1%	83.4%	82.3%	82.2%	82.9%	**85.6%**
	Several days	**11.6%**	10.9%	11.0%	9.8%	9.7%	8.5%
	More than half the days	2.6%	3.0%	3.2%	**4.0%**	3.6%	2.6%
	Nearly every day	2.7%	2.6%	3.5%	**4.0%**	3.8%	3.3%
Psychomotor	Not at all	84.4%	**90.4%**	89.3%	87.7%	87.7%	90.0%
	Several days	**7.4%**	6.3%	6.8%	7.1%	6.3%	5.6%
	More than half the days	1.1%	1.8%	1.8%	**3.2%**	3.0%	2.0%
	Nearly every day	1.4%	1.5%	2.1%	2.0%	**3.0%**	2.4%
Suicidal ideation	Not at all	96.4%	**97.0%**	96.6%	95.5%	96.0%	96.3%
	Several days	2.6%	1.8%	2.4%	**2.7%**	2.5%	2.3%
	More than half the days	0.6%	0.7%	0.6%	**0.8%**	0.5%	0.7%
	Nearly every day	0.4%	0.5%	0.4%	**1.0%**	1.0%	0.7%
Mean probability of each response option	Not at all	75.3%	76.8%	76.6%	75.1%	76.3%	**79.7%**
	Several days	**17.1%**	15.6%	15.3%	14.6%	14.1%	12.2%
	More than half the days	4.1%	4.0%	3.8%	**4.9%**	4.4%	3.4%
	Nearly every day	2.9%	3.6%	4.3%	**5.5%**	5.1%	4.7%

The highest probabilities of each response option among all age groups are in bold.

### Age-Related Changes in the Probability of Responses for the Nine Items

**Figure 5:**
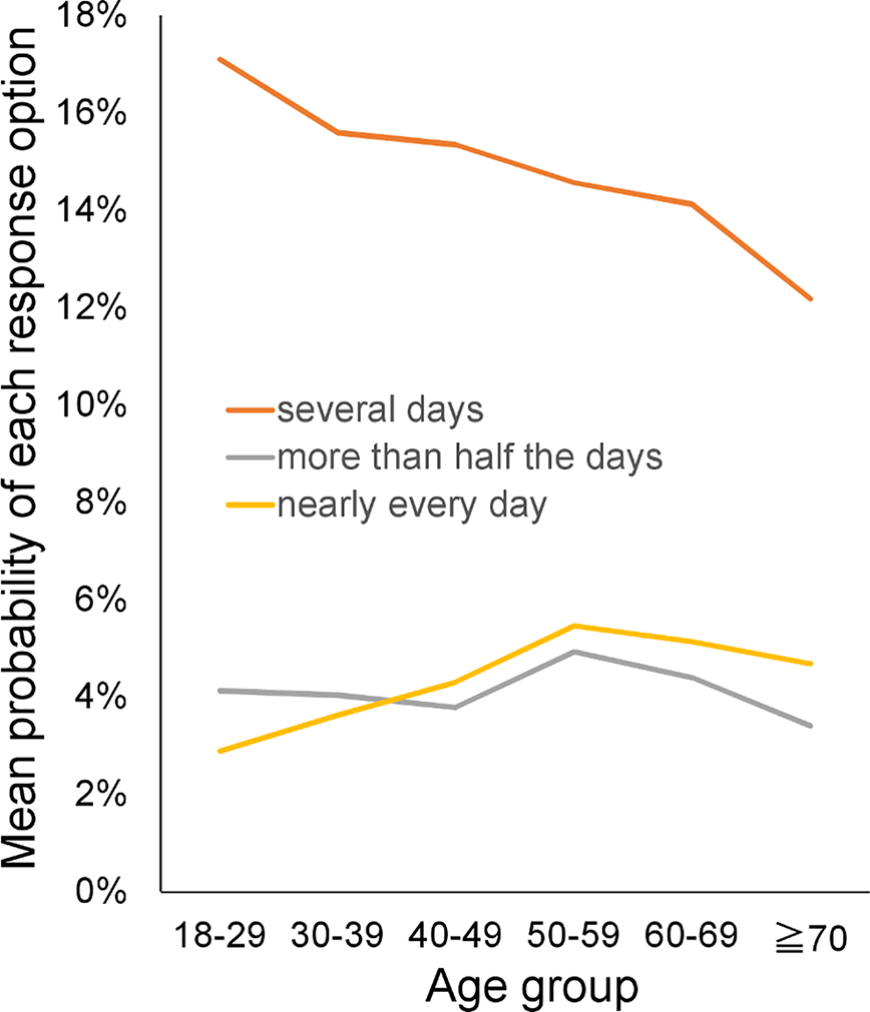
Age-related changes in the mean probability of each response option. The mean probability of a response of “several days” decreased with age. Conversely, the mean probability of responses of “more than half the days” and “nearly every day” exhibited a reverse U-shaped pattern that peaked at 50–59 years.

To illustrate age-related changes in item responses, we calculated the mean probability of responses of “several days,” “almost half the days,” and “nearly every day” for the nine items and demonstrated the trajectory of the mean probability for each item option (**Figure 5** and **Table 2**). The mean probability of a response of “several days” decreased with age. Conversely, that of responses of “more than half the days” and “nearly every day” exhibited a reverse U-shaped pattern, peaking at 50–59 years.

The total PHQ-9 score and most item scores exhibited a reverse U-shaped pattern across adulthood, increasing from 18–29 to 50–59 years and decreasing at ≥60 years (**Figure 2** and **Table 1**). These trajectories could be explained by age-related changes in the probability of each item response option.

As shown in **Figure 5**, while the mean probability of a response of “several days” decreased between 18–29 and 50–59 years (from 17.1% to 14.6%), that of responses of “more than half the days” and “nearly every day” increased during the same period (from 4.1% to 4.9% and from 2.9% to 5.5%, respectively) (**Table 2**). Although the decrease in the probability of responses of “several days” was greater than the sum of the increases in the probability values for responses of “more than half the days” and “nearly every day,” “more than half the days” is doubled and “nearly every day” is tripled in the calculation of mean item scores. As a result, the total PHQ and item scores increased between 18–29 and 50–59 years (**Figure 2** and **Table 1**). Conversely, the mean probability values for responses of “several days,” “more than half the days,” and “nearly every day” decreased between 50–59 years and ≥70 years (**Figure 5**). Therefore, total PHQ and item scores decreased at 50–59 years (**Figure 2** and **Table 1**), showing a reverse U-shaped pattern across adulthood.

## Discussion

The aim of this study was to determine whether the item responses for the PHQ-9 followed the same characteristic pattern across adulthood and elucidate whether the item response pattern contributed to age-related changes in total scores.

The main findings of the study were as follows: (1) the characteristic pattern of the item responses was confirmed across the adult lifespan; (2) the total PHQ-9 score and most of the item scores exhibited similar reverse U-shaped patterns across adulthood; and (3) while the average probability of a response of “several days” decreased with age, that for “more than half the days” and “nearly every day “ exhibited a reverse U-shaped pattern across adulthood.

The present study demonstrated that PHQ-9 item responses followed the same mathematical pattern across adulthood. In accordance with these findings, the results of previous research showed that item responses for the CES-D followed a characteristic pattern across adulthood (26), indicating that item responses in depression symptom scales maintain a characteristic distribution across the adult lifespan. It is important to understand how this mathematical pattern of item responses emerges in the general population. Response options on depression symptom scales begin with absence at the lower end (e.g., not at all) and follow with symptom severity (e.g., several days, more than half the days, and nearly every day on the PHQ-9) (30). According to the findings of previous studies, this characteristic pattern emerges when the ratios of “several days” to “more than half the days,” and “more than half the days” to “nearly every day,” are similar among all items. (**Figure 1**) (19, 21, 31). Therefore, further research involving a greater focus on the similarity of ratios of “several days” to “more than half the days,” and “more than half the days” to “nearly every day,” for all items is required.

Statistical models that assume normal distributions are often used to assess the psychometrics of depression rating scales. However, our results provide further evidence that the pattern of responses to depressive symptom items differs from a normal distribution in a general population. These findings suggest that statistical models assuming normality might require consideration in analyzing responses to depressive symptom items.

The present findings indicated that reverse U-shaped patterns in total PHQ-9 and item scores were attributed mainly to age-related changes in the probability of a response of “nearly every day,” which was the highest score option for severity words. These results are consistent with those of a previous study indicating that total CES-D scores and the probability of a response of “all the time” (the highest score option on the CES-D scale) exhibited a similar U-shaped pattern across adulthood (26). It should be noted that the trajectory of the probability of the most severe response differed between the present (reverse U-shaped) and previous studies (U-shaped) (26). Further studies are required to clarify the factors that determine the trajectory of the probability value for the highest score option.

As stated previously, the decrease in the probability of a response of “several days” was greater than the sum of the decreases in the probability of responses of “more than half the days” and “nearly every day” between 18–29 and 50–59 years. These results raise the possibility that differences in scoring methods, such as that between binary and four-point scales, could result in different age-related patterns across adulthood. When the mean probability values for responses of “several days,” “more than half the days,” and “nearly every day” for the nine items are presented as P_1_, P_2_, and P_3_, respectively. On an ordinal scale (i.e., 0-1-2-3), the mean item scores for the nine items were expressed as P_1_ + 2P_2_ + 3P_3_. If we apply a binary scale (0-1) that lacks “more than half the days” and “nearly every day” response options to the present data, the mean item score for the nine items would be P_1_ + P_2_ + P_3_. As the decrease in P_1_ is greater than the sum of the increases in P_2_ and P_3_ from 18–29 to 50–59 years, total PHQ-9 and item scores on the hypothetical binary scale would decrease across adulthood. In support of this speculation, previous research using binary depression symptom scales, such as the Goldberg Depression Scale (32), has demonstrated a downward trajectory in total scores across adulthood (33). Future studies should focus on identifying the effects of scoring methods on age-related changes in total scores on depression symptom scales.

The study involved methodological strengths and limitations. For example, the data were representative of the US population with data traceability. Further, the large sample size (*N* = 15,431) allowed us to identify patterns in item responses. In addition, we used graphical analysis to demonstrate the pattern in item responses. In general, graphical analysis is a useful method for identifying complex patterns (34). Conversely, although we demonstrated that item responses for the PHQ-9 followed the same model during adulthood, we did not quantify the fitness of the model for all item responses, because of the complexity of the pattern. Generally, the fitness of a unitary model can be quantified *via* regression analysis. However, it is difficult to quantify the fitness of a complex model. Further research is required to develop a method *via* which to quantify the fitness of the complex pattern observed in this study.

In conclusion, this study demonstrated that PHQ-9 item responses in the general population followed a similar characteristic pattern across the adult lifespan, and the trajectory of total PHQ-9 scores was attributed mainly to age-related changes in the probability of the occurrence of the highest score option in the PHQ-9 scale. The specific pattern of item scores observed in this study could contribute to our understanding of age-related changes in item responses for depressive symptoms and help predict how the degree of depressive symptoms is distributed in a general population. Future research should focus on the mechanisms *via* which item responses in depressive symptom scales exhibit a characteristic pattern.

## Data Availability Statement

Publicly available datasets were analyzed in this study. This data can be found here: https://www.cdc.gov/nchs/nhanes/index.htm.

## Author Contributions

ST designed the study and was responsible for the acquisition and analysis of data. YK, KI, MA, and ST were involved in the interpretation of the data and preparation of the manuscript. ST was a major contributor to the writing of the manuscript. TF and YO were involved in the study design and preparation of the manuscript. All authors contributed to the article and approved the submitted version.

## Funding

This work was supported by a research grant from the JSPS KAKENHI (grant number 18K03145). The funding source had no role in designing the study, in the collection, analysis and interpretation of data; in the writing of the report; or in the decision to submit the article for publication.

## Conflict of Interest

The authors declare that the research was conducted in the absence of any commercial or financial relationships that could be construed as a potential conflict of interest.
