# Supplementary material for: Age-Related Changes in Item Responses to the Patient Health Questionnaire-9: Evidence From the National Health and Nutrition Examination Survey
Source: Front Psychiatry. 2020 Jul 22;11:723. doi: 10.3389/fpsyt.2020.00723 (PMC7387704; doi:10.3389/fpsyt.2020.00723)
Supplement: Supplementary file 1 [file Table_1.docx]

**PATIENT HEALTH QUESTIONNAIRE-9 (PHQ-9)**

|  | **Over the last 2 weeks, how often have you been bothered by any of the following problems?** | **Not at all** | **Several days** | **More than half the days** | **Nearly every day** |
| --- | --- | --- | --- | --- | --- |
| **1.** | Little interest or pleasure in doing things | 0 | 1 | 2 | 3 |
| **2** | Feeling down, depressed, or hopeless | 0 | 1 | 2 | 3 |
| **3.** | Trouble falling or staying asleep, or sleeping too much | 0 | 1 | 2 | 3 |
| **4.** | Feeling tired or having little energy | 0 | 1 | 2 | 3 |
| **5.** | Poor appetite or overeating | 0 | 1 | 2 | 3 |
| **6.** | Feeling bad about yourself — or that you are a failure or have let yourself or your family down | 0 | 1 | 2 | 3 |
| **7.** | Trouble concentrating on things, such as reading the newspaper or watching television | 0 | 1 | 2 | 3 |
| **8.** | Moving or speaking so slowly that other people could have noticed? Or the opposite — being so fidgety or restless that you have been moving around a lot more than usual | 0 | 1 | 2 | 3 |
| **9.** | Thoughts that you would be better off dead or of hurting yourself in some way | 0 | 1 | 2 | 3 |
